# Popliteal Pterygium With Van Der Woude Syndrome

**DOI:** 10.7759/cureus.16573

**Published:** 2021-07-22

**Authors:** Monica Dobs, Mohamed A Ghamry, Priyanka Anvekar, Petras Lohana, Syed R Ali

**Affiliations:** 1 Pediatrics and Neonatology, Assiut University, Faculty of Medicine, Assiut, EGY; 2 Pediatrics, Al-Salam Hospital, Ministry of Health, Port Said, EGY; 3 Pediatrics, Mahatma Gandhi Mission Medical College and Hospital, Mumbai, IND; 4 Internal Medicine, Liaquat University of Medical and Health Sciences Hospital, Karachi, PAK; 5 Internal Medicine, Dow University of Health Sciences, Civil Hospital Karachi, Karachi, PAK

**Keywords:** dysmorphic features, vw syndrome, irf6 gene, popliteal pterygium syndrome, fasciogenito-popliteal syndrome, popliteal web syndrome

## Abstract

Van der Woude syndrome (VWS) is an autosomal dominant syndrome due to mutation of a gene located in the long arm of chromosome 1 (1q32.3-q4) called *the interferon regulatory factor-6* (IRF6) gene. VW syndrome-affected children are born with a cleft lip or palate, hypodontia (absent teeth), and bilateral paramedian lower-lip pits, which are usually moist because they are often associated with accessory salivary glands and mucous glands that empty into the pits. Popliteal pterygium syndrome (PPS), also known as a fasciogenito-popliteal syndrome or popliteal web syndrome is a rare autosomal dominant disorder with an incidence of approximately 1 in 300,000 live births. The most common clinical manifestations are popliteal webbing, cleft palate, cleft lip, syndactyly, and genital and nail anomalies. This report describes the clinical features in one case with positive family history, showing the range of anomalies found in popliteal pterygium with VWS.

## Introduction

Van der Woude syndrome (VWS) is present in about 2% of all patients with cleft lip and/or palate with a prevalence of approximately 3.6/100,000 of live births. The bilateral, paramedian lower lip pits with or without cleft palate and/or lip is typical of this syndrome Patients commonly present with cleft lip, cleft lip, and palate or with cleft palate only. Popliteal pterygium syndrome (PPS) shares some features with VWS, but, in addition, has a characteristic cutaneous webbing or skin fold that extends from the ischium to the heel (“popliteal pterygium”), associated with toenail dysplasia, eventual foot and toes deformities, oral cavity, and genitourinary anomalies. VWS and PPS are autosomal dominantly inherited disorders caused by heterozygous mutations in the interferon regulatory factor-6 (IRF6) gene. In 68% of patients with VWS and 97% of patients with PPS, a heterozygous IRF6 mutation is detectable. We reported a case of a girl who has a manifestation of both syndromes which could probably support the hypothesis that both syndromes could represent variants of the same condition [1].

## Case presentation

**Figure 1:**
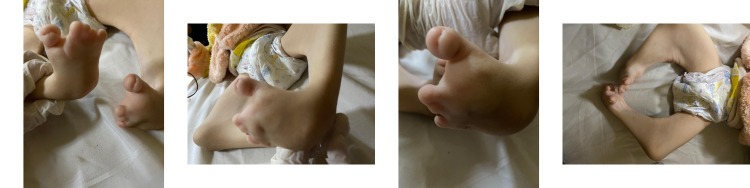
Lower extremities deformities in the patient with Van der Woude syndrome.

**Figure 2:**
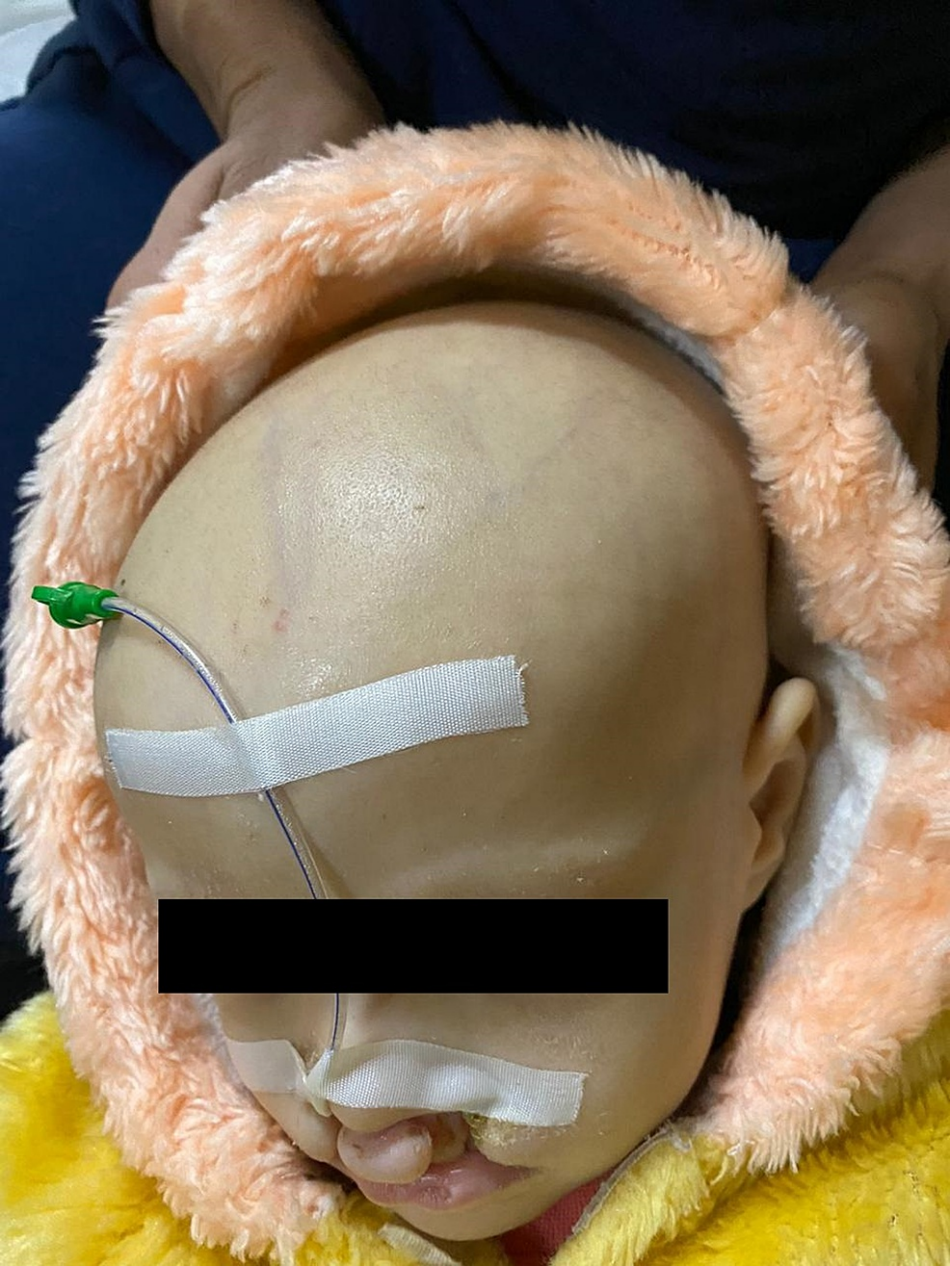
Alopecia totalis and presence of nasogastric tube in the patient due to feeding difficulties.

A five-year-old female patient presented to the pediatric emergency department with severe pallor. The patient was born full-term, normal vaginal delivery, conceived out of a consanguineous marriage. The patient has no history of NICU admissions in the past. Family history is positive for death in her sibling at the age of 3 due to pneumonia. There is also a history of no healthy living children. The patient’s parents decided to have no children in the future. On physical examination, she had deformities in both lower extremities and joints (Figure 1) and alopecia totalis (Figure 2).

**Figure 3:**
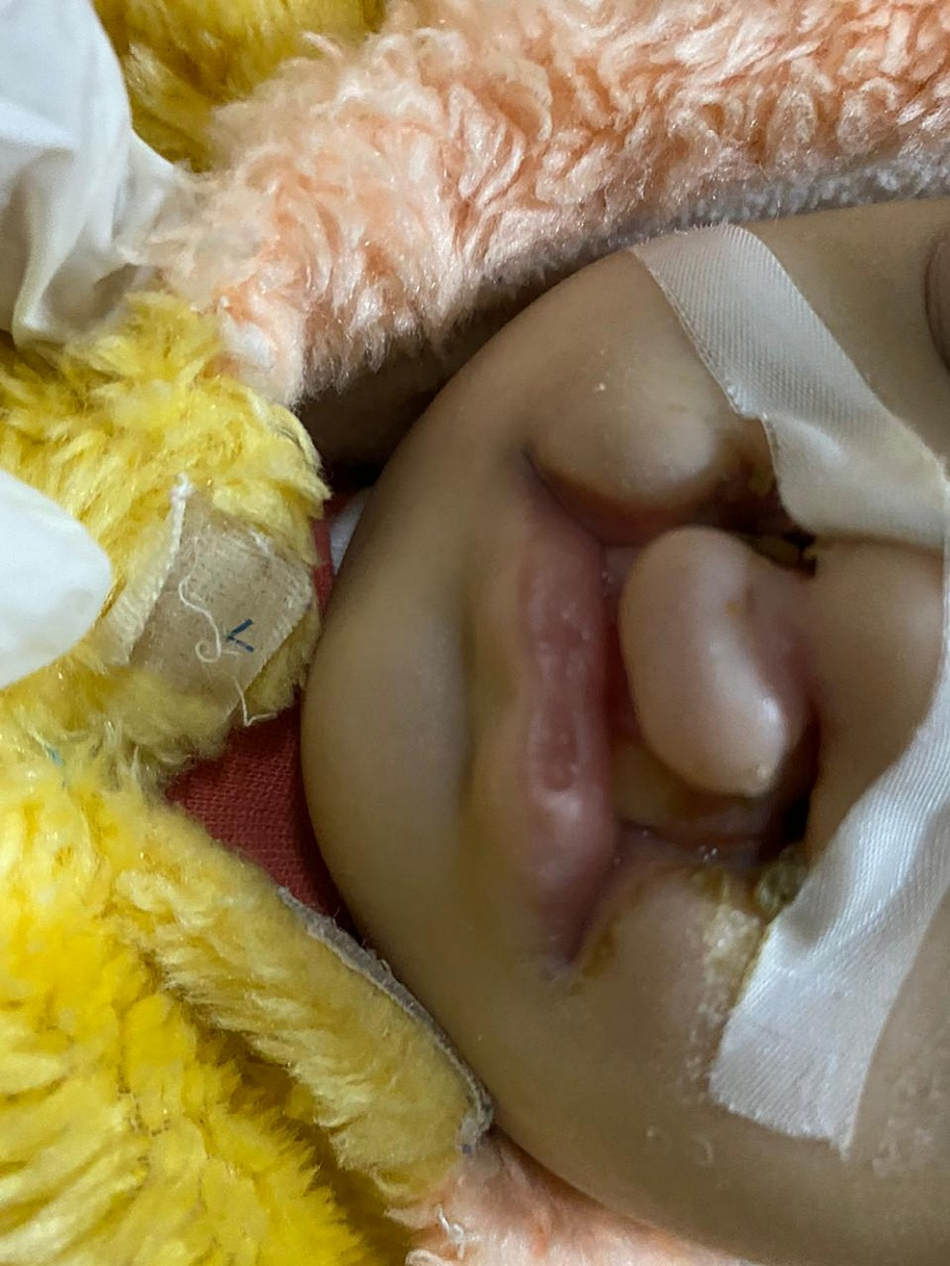
Cleft lip and cleft palate in the patient.

She also had a cleft lip and cleft palate and there was a presence of nasogastric tube due to feeding difficulties because of her cleft palate and cleft lip (Figure 3). The patient was immediately admitted to the pediatric ward. Laboratory investigations on admission were as follows: hemoglobin (Hb): 4.2 g/dL (9.5-14 g/dL), red blood cells (RBC): 3 million cells/microlitre (3.5-5 million cells/microlitre), white blood cells (WBC): 7 x 10^9^/litre (5-10 x 10^9^/litre), platelet count: 584 x 10^9^/litre (205-483 x 10^9^/litre). Further investigation revealed mean corpuscular hemoglobin concentration (MCHC) 220 g/litre (320-360 g/litre), mean cell value (MCV) of 61.0 fL (74.5-93.9 fL), serum iron 2.1 μmol/L (8-24 μmol/L), serum total iron-binding capacity (TIBC) of 93.8 μmol/L (44-85 μmol/L), transferrin saturation of 23% (0.12-0.46%) and ferritin of 2.3 μg/L (12-270 μg/L), red cell distribution width (RDW) of 17% (11.6-14.8%). A review of other systems was within normal limits. Electrolytes, liver profile, bilirubin, and renal profile were normal. There were no signs of hemorrhage or hemolysis detected. The patient’s blood was sent for cross-matching and an arrangement for packed RBC for transfusion was made. Written consent was obtained from the parent and the patient was given 15mL/Kg of packed RBCs along with oxygen as supportive therapy. The patient tolerated the treatment well and was successfully stabilized. The patient’s Hb was rechecked after the treatment and it was 9.6 g/dL. She was discharged with the advice of regular follow-ups, dietary advice, and timely investigations. The parents were counseled about the patient’s prognosis and referred to speech therapy.

## Discussion

PPS is a rare autosomal dominant disorder with an approximate incidence of 1 in 300,000 live births and shows both inter-and intrafamilial variation [2]. The term PPS was given by Gorlin in 1968 and was first described by Trelat in 1869 [3]. The percentage of presence of clinical manifestations are cleft palate (93%), popliteal webbing (58%), cleft lip (58%), lower lip pits (46%), syndactyly (50%), genital anomalies (37%), and nail anomalies (33%). Other rare reported clinical features include talipes, ankyloblepharon, syngnathia, and digital reduction defects. IQ is usually normal 2 but there is a risk of delayed language development, learning disabilities, and other mild cognitive problems [4]. PPS has a highly variable expression and incomplete penetrance. Genetic heterogeneity of environmental factors cannot be excluded in sporadic occurrences [3].

VWS is present in approximately 2% of all cleft patients. The bilateral paramedian lower lip pits with or without cleft lip and/or palate is typical of this syndrome and the pits have sinuses from salivary and mucous glands so they are usually discharged. Hypodontia is not rare in VWS, while single median salivary lower lip pit and unique single big tooth are rarely reported manifestations [3].

PPS and VWS are allelic variants of the same condition because they are caused by different mutations of the same gene. PPS includes all the features of VWS, plus popliteal pterygium, syndactyly, distinct toe/nail abnormality, syngnathia, and genitourinary malformations [5].

The cause of VWS is a mutation in the IRF6 gene of the long arm of chromosome 1 (1q32.3-q4). The gene is responsible for making a protein that plays an important role in early development and is active in cells that give rise to tissues in the head, face, skin, and genitals. The PPS is associated also with mutations in the IRF6 (1q32.2-q32.3) gene, involved in the formation of epithelial and connective tissues. Prognosis is depending on the severity of pterygium and genital anomalies may cause infertility otherwise prognosis of growth and intelligence is good [4].

Cleft lip and/or palate should be treated surgically at an early stage. Speech therapy, as well as audiological and dental assessments, should also be provided. The choice of treatment method for popliteal pterygium of PPS may be difficult, but in general surgical treatment is preferred because the conservative treatments, including serial casting or traction, have unsatisfactory outcomes. Among these surgical treatments, resection of fibrous bands and Z-plasty lead to a lengthening of the soft tissues such as the skin, muscles, and ligaments were the most preferred surgery. However, If the sciatic nerve is displaced into the webbing and it is attached to the fibrous tissues, this surgery will not be applicable and nerve grafting may be additionally needed. In severe cases, amputation may be the only option. Correction of deformities might be obtained with knee arthrodesis, femoral shortening, or femoral extension osteotomy [6]. 

## Conclusions

This case of VWS and PPS is reported for its rarity and the high importance of detecting its features and support the affected children with suitable nutritional, medical and surgical treatment to improve their quality of life and health. Both syndromes have autosomal dominant inheritance and both have a mutation in gene IRF6. Both syndromes share common clinical manifestations as cleft lips and palate, pits on lower lips and palate, salivary gland and speaking problems, and others. PPS has an affection for extremities and external genitalia. Some anomalies especially face anomalies can be diagnosed early, in utero with ultrasound. Today, the prognosis and treatment of individuals with VWS are excellent. The prognosis of individuals with PPS will depend on severity.
